# Accurate Diagnosis of Suicide Ideation/Behavior Using Robust Ensemble Machine Learning: A University Student Population in the Middle East and North Africa (MENA) Region

**DOI:** 10.3390/diagnostics10110956

**Published:** 2020-11-16

**Authors:** Azam Naghavi, Tobias Teismann, Zahra Asgari, Mohammad Reza Mohebbian, Marjan Mansourian, Miguel Ángel Mañanas

**Affiliations:** 1 Department of Counseling, Faculty of Education and Psychology, University of Isfahan, Azadi Sq, Isfahan 8174673441, Iran; 2 Department of Clinical Psychology and Psychotherapy, Ruhr-Universität Bochum, 44787 Bochum, Germany; tobias.teismann@ruhr-uni-bochum.de; 3 Department of Counseling, Faculty of Education and Psychology, University of Isfahan, Isfahan 8174673441, Iran; za.asgari@edu.ui.ac.ir; 4 Department of Electrical and Computer Engineering, University of Saskatchewan, Saskatoon, SK S7N5A9, Canada; mom158@usask.ca; 5 Biomedical Engineering Research Centre (CREB), Automatic Control Department (ESAII), Universitat Politècnica de Catalunya-Barcelona Tech (UPC), 08028 Barcelona, Spain; miguel.angel.mananas@upc.edu; 6 Epidemiology and Biostatistics Department, Health School, Isfahan University of Medical Sciences, Isfahan 81746-73461, Iran; 7 Biomedical Research Networking Center in Bioengineering, Biomaterials, and Nanomedicine (CIBER-BBN), 28029 Madrid, Spain

**Keywords:** suicide, traumatic events, screening tool, university students, machine learning, Middle East and North Africa (MENA)

## Abstract

Suicide is one of the most critical public health concerns in the world and the second cause of death among young people in many countries. However, to date, no study can diagnose suicide ideation/behavior among university students in the Middle East and North Africa (MENA) region using a machine learning approach. Therefore, stability feature selection and stacked ensembled decision trees were employed in this classification problem. A total of 573 university students responded to a battery of questionnaires. Three-fold cross-validation with a variety of performance indices was sued. The proposed diagnostic system had excellent balanced diagnosis accuracy (AUC = 0.90 [CI 95%: 0.86–0.93]) with a high correlation between predicted and observed class labels, fair discriminant power, and excellent class labeling agreement rate. Results showed that 23 items out of all items could accurately diagnose suicide ideation/behavior. These items were psychological problems and how to experience trauma, from the demographic variables, nine items from Post-Traumatic Stress Disorder Checklist (PCL-5), two items from Post Traumatic Growth (PTG), two items from the Patient Health Questionnaire (PHQ), six items from the Positive Mental Health (PMH) questionnaire, and one item related to social support. Such features could be used as a screening tool to identify young adults who are at risk of suicide ideation/behavior.

## 1. Introduction

According to the World Health Organization’s (WHO) report [1], the rate of death by suicide is close to 800,000 globally. Suicide, as one of the most critical public health concerns, is the second reason for death among young people between the ages of 15 to 29 around the world. This age group in many countries belong to high school and university students. In England, the rate of university students’ suicide has increased since 2009 [2], and, in the USA, death by suicide was ranked second among the same age group [3]. Suicide is more prevalent in developing countries, and, according to global statistics, around 79% of the annual suicide rate belongs to developing countries [1]. Despite some claims that suicide rate is lower in countries with dominant religious culture, a recent study including 12 countries with high Muslim population in Asia, Middle East, and Africa, found that the rate of university student suicide in these countries is comparable to the global data. Iran, as one of the developing countries located in the Middle East and North Africa (MENA) region, followed the global trend of suicide among university students. The university student suicide rates in Iran for men, women, and both were 4.20%, 2.90%, and 3.60%, respectively [4]. In one epidemiological survey of attempted suicide in Iran during 2007–2011, 72% were below 30 years [5]. In another study, the outcome and varieties of violent suicides during 20 years in the Southwest of Iran were investigated, and the age group 25–34 years were found to have the highest number of attempted suicides [6]. Bakhtar and Rezaeian [7] found that the rate of suicide attempts in the Iranian population was between 1.8% to 3.5%, and for suicide ideation between 6.2% to 42.7%. 

As one of the leading causes of death for young people, suicide, and measures to prevent it are among the most critical health priorities in the “Mental Health Action Plan 2013–2020” [8]. To prevent suicide, researchers from different disciplines have tried to explore its predictors. Social and economic factors such as marital conflicts [5], a romantic breakup [9,10], and unemployment [11], are associated with suicide ideation and behavior. Yet, psychological issues are often considered as the leading reasons for suicide. Depression is strongly related to suicide in many studies [5,12,13,14].

Exposure to trauma and post-traumatic stress disorder (PTSD) is yet another psychological issue that is significantly associated with suicide [15,16,17,18]. Trauma is defined as a deeply distressing and unpredictable event, which can occur directly or indirectly to individuals, and has ongoing adverse aftermaths on the person’s physical, social, emotional, or psychological functioning [19]. The most common age for exposure to traumatic events is 16 to 20 [20] that has an overlap with the typical age group for suicide ideation and behavior. Repeated trauma exposure increases the risk of suicide behavior, and this finding is true for both natural and human-made traumatic events [21]. In the WHO mental health survey [22], 102,245 households in 21 countries reported a range of traumatic events that were associated with suicidal behaviors. The most prevalent types of traumatic events across both developed and developing countries were death of a loved one, witnessing of interpersonal violence, war, accidents, or traumatic event for a loved one. In this study, 9.6% of the population reported suicide ideation, and 2.8% had suicide attempts. Human-made trauma such as sexual and interpersonal violence had the most substantial effect on suicide behavior, and the number of traumatic events influences on the suicide ideation or attempt.

A time gap between suicide ideation and suicide attempt is critical in mental healthcare. Many researchers found a relationship between experiencing a traumatic event and post-traumatic stress disorder (PTSD) with higher risk of suicide ideation, and there is an association between the number and type of traumatic events and suicidality [22]. Several factors can protect people from suicidal behaviors. Positive mental health (PMH) [23] is one of these protecting factors. Some recent studies found that people with positive mental health (psychological and subjective well-being) are less likely to attempt suicide, even if they have suicide ideation [24,25,26,27,28]. Teismann and colleagues [29] found a moderating effect of PMH on the association of depression with suicidal thoughts among university students. Post-traumatic growth (PTG) can also affect suicide ideation and behaviors after a traumatic event [30,31,32]. Post-traumatic growth, as Tedeschi and Calhoun [33] defined it, refers to significant positive psychological changes in cognitive and emotional life resulting from the struggle with new conditions following traumatic or extremely stressful events. In particular, there are five domains of PTG: a greater appreciation of life, stronger social connections, developing a sense of power, recognizing few life’s opportunities, and more spirituality [34]. People who experience PTG may be less likely to attempt suicide. Other factors, such as social support [30], resilience [35], and cognitive distortion [36], have been also shown to affect the relationship between suicide ideation and a suicide plan/attempt. 

Although some studies attempted to predict the risk of suicide by investigating risk and protective factors, there is little knowledge of clinical prediction rules to predict university students’ suicide ideation/behavior. Since suicide is a multi-faceted phenomenon that has complex risk and protecting factors to predict suicide ideation/ behavior, we need to use algorithms that “model complex relationships among a large number of factors” [37]. Recently, machine learning (ML) and related techniques started to enter the field of psychiatry and psychology with regard to suicide [38,39]. Passes and colleagues [17] predicted suicide in a sample of people with a mood disorder and found that previous hospitalization for major depression, PTSD, drug dependence, and psychosis could strongly predict the risk of death by suicide. In another study, Kessler and colleagues [40] predicted suicide in a sample of soldiers and veterans and found male sex, criminal offenses, and previous suicide ideation as the most influential risk factors for completed suicide. Kuroki [41] used a data mining approach to predict suicidal behaviors among 624 Filipino Americans and found depression and substance use disorder as the critical predictors of suicidal ideation. This study, however, did not focus on the sensitivity and specificity of the proposed diagnosis system. DelPozo-Banos and colleagues [13] explored the feasibility of using artificial neural networks to identify suicide risk among 2604 completed suicide cases. Prescription of psychotropic, depression, anxiety, and self-harm increased the risk of suicide in this system. The sensitivity, specificity, and accuracy of the proposed system were 64.57%, 81.86%, and 73.22%, respectively. Walsh and colleagues [42] utilized the ML approach to predict a suicide attempt among adolescents under the age of 18. Data used for this analysis extracted from electronic health records between 1998 to 2015 from a single medical center. The AUC in this system was 0.9. However, the sample group (adolescents) was different from the current study. In another study, Walsh and colleagues [43] conducted the same study among the adult population and found that the ML approach could predict the risk of suicide with an accuracy of 0.84. Both studies focused on a clinical sample that is different from the target group of the current study. Another study that can be comparable to our study is Ribeiro and colleagues [44]. Although it showed a good performance (AUC: 0.9), the population was different (adults with a history of self-injury), and it did not focus on important psychological variables that have been shown to be related to suicide, such as positive mental health, post-traumatic stress disorder, and post-traumatic growth. Apparently, the existing studies that used big data to predict suicide, used a limited number of inputs or omitted psychologically important inputs such as PTSD, while, as mentioned before, experiencing traumatic events and suicide risk is correlated. Moreover, there is an overlap between the age of trauma exposure [20], and suicide risk age [1] among university students. To the best of our knowledge, there is no study to diagnose suicide ideation/ behavior among university students in the MENA region and likely in the world with the help of machine learning. 

Moreover, a reliable medical diagnosis system must meet the following simultaneous conditions, namely sensitivity, specificity, positive predictive value, and diagnostic odds ratio, higher than 80%, 95% [45], 95% [46], and 100% [47]. The methods developed in the literature do not meet such conditions. Therefore, this study aimed to diagnose suicide ideation/behavior through a machine learning approach toward a clinically-reliable diagnosis.

## 2. Materials and Methods

### 2.1. Sample Size Calculation and Sampling 

Assuming the suicide ideation prevalence of (*p* = 26%) [48], the total sample size (*N*) with the precision of (*d* = 5%) and a type I error of (*α* = 0.01) could be calculated as the following [49,50].

(1) $$N=\frac{z_{1-\alpha /2}^{2}\times p\times \left(1-p\right)}{d^{2}}$$

A total of 511 subjects were sufficient. We have used an online sampling method to recruit the participants. This method is suggested to be an efficient, affordable, and practical method of sampling in national and international sampling [51], and is used in a number of studies with the student population [52,53,54]. A link for the questionnaire was created through Google form, and was distributed through different platforms including the universities’ WhatsApp, Telegram, or Instagram groups. The recruitment process began in March 2020 and was stopped in May 2020 when we reached enough completed questionnaires, and, finally, 573 questionnaires with complete information were analyzed. Demographic characteristics consisted of age, gender, grade and educational level, the field of study, marital and occupational status, and history of psychological illness diagnosis. Moreover, the following six questionnaires were used.

### 2.2. PTSD Checklist (PCL-5)

The PTSD Checklist (PCL-5) [55] is a self-report checklist. This checklist has 20 items and screens the symptoms of PTSD and their severity in the last month. The checklist determines the most prevalent symptoms of PTSD including intrusion, avoidance, cognitive and mood alteration, and arousal and reactivity alteration in a 0 (not at all) to 4 (extremely) Likert scale. The scores range between 0 to 80, and 31 is considered as the cut-off score. People who scores 31 or higher in this scale indicate more PTSD symptoms with a specificity, sensitivity, and efficiency of 0.95, 0.85, and 0.95, respectively [56]. In this study, the Iranian version of the PCL-5 was used [57]. A 0.939 Cronbach’s alpha was found for the current sample.

### 2.3. Post-Traumatic Growth Inventory (PTGI)

The Post-Traumatic Growth Inventory (PTGI) [58] assesses growth after traumatic events. This inventory has 21-items that focus on the five domains that may positively change after traumatic events. These domains include more and greater social connections, new possibilities, higher perceived skills and resources, more life’s appreciation, and strengthening spiritual beliefs. The inventory is evaluated on a 6-point Likert scale (0–5) and higher scores demonstrate more PTG. Scores range between 0 to 105 with a cut-off point of 45, whereas the higher score show higher growth [59]. The PTGI has well-established validity and reliability, with Cronbach’s alpha values between 0.67 and 0.90 [58]. In this study, the Iranian version of the PTGI was used [60]. The Cronbach’s alpha for the current sample was 0.937.

### 2.4. Patient Health Questionnaire (PHQ-9)

The Patient Health Questionnaire (PHQ-9) [61] is yet another self-report questionnaire with nine items. This questionnaire looks at the frequency of the nine major depressive symptoms in the past two weeks. The Likert scale ranging from 0 to 3 (not at all- nearly every day) score shows a mild (scores 5–9), moderate (10–14), moderately severe (15–19), and severe (20–27) depression. With a cut-off score of 10, the scale can detect major depression with sensitivity and specificity of 88% and 88%, respectively [62]. In this study, the Iranian version of the PHQ-9 was used [63]. The Cronbach’s alpha for the current sample was 0.879.

### 2.5. Multidimensional Scale of Perceived Social Support (MSPSS)

“Family, Friends, and Significant Others” are the three domains of perceived social support that are assessed by the Multidimensional Scale of Perceived Social Support (MSPSS), as a self-report scale [64]. A 6-point Likert scale (1–7) assesses the level of perceived social support, and the total score ranges between 12 to 84 with a cut-off point of 48. The higher scores show a higher sense of having social support. In this study, the Iranian version of the MSPSS with good validity and reliability was used [65]. The Cronbach’s alpha for the current sample was 0.922.

### 2.6. Positive Mental Health Scale (PMH)

The Positive Mental Health Scale (PMH) [66] assesses emotional and psychological aspects of well-being using nine items. Each item scores between 0 to 3 (do not agree-agree) and the higher scores show higher PMH with a cut-off point of 15. The scale showed good validity in different populations. In this study, the Iranian version of the PMH was used [67]. In the current sample, the Cronbach’s alpha was 0.905.

The cut-off values for PMH and MSPSS questionnaire were calculated using the cut-off estimation method for receiver operating characteristic curve (ROC) proposed by Unal [68]. In this method, the “optimal” cut-point (c) is defined as the point to minimize the error function defined below.

(2) $$\mathrm{ER}\left(c\right)=\sqrt{{\left(1-\mathrm{Se}\left(c\right)\right)}^{2}+{\left(1-\mathrm{Sp}\left(c\right)\right)}^{2}}$$

where Se and Sp are the sensitivity and specificity obtained when comparing the results with the gold standard (high and low suicide risk).

Thus, the cut-off values were estimated as 15 and 48 for PMH and MSPSS, respectively. The area under the ROC Curve (AUC) were 0.743 [CI 95%: 0.695, 0.790], and 0.661 [CI 95%: 0.609, 0.713], for PMH and MSPSS, respectively. 

### 2.7. Suicide Behaviors Questionnaire-Revised (SBQ-R)

The Suicide Behaviors Questionnaire-Revised (SBQ-R) [69] contains four multiple-choice items that assess the frequency and severity of suicide ideation, suicidal attempts in the past year, and the possibility of suicide behavior in the future. The total SBQ-R severity scores range from 3 to 18 and the cut-off score of ≥8 can identify high and low risk groups. In addition, the cut-off score for the first question is ≥2. The Cronbach’s alpha of 0.76 and 0.88 in nonclinical and clinical samples was reported in Osman et al. [69]. In this study, the Iranian version of the SBQ-R was used [70]. For the current sample, the Cronbach’s alpha was 0.828.

### 2.8. Ethical Considerations

Ethics approval of the research was granted by the University of Isfahan’s Ethic Committee with the number IR.UI.REC.1399.008 (approval date: 15 February 2020). To ensure confidentiality, the questionnaires were anonymous. Respondents could refrain from answering anytime they wished. There was no incentive for participation. However, participants could write an email and receive their test interpretations.

### 2.9. Methods

Choosing informative, discriminating, and stable features is a crucial step for designing a classification model [71]. In this research, stability feature selection is performed [72]. The idea of stability selection is to inject more noise into the original problem by providing bootstrap data batches and to use a baseline feature selection algorithm to investigate which features are essential in each sampled version of the data. The results of each bootstrap sample are then used to calculate the stability score for each feature. For this purpose, logistic regression [73] is utilized to analyze feature importance in each bootstrap step, and, if a feature was significant in most of the iterations, it would be selected as a stable feature. Such a selection was made based on the Type one error of 0.05 or less.

**Figure 1:**
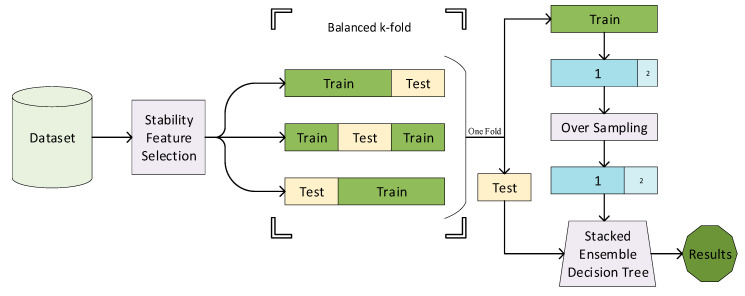
The block diagram of the proposed method. The features are first selected using stability feature selection. Using the stratified sampling, the features are then classified using a stacked ensemble decision tree.

Ensemble learning is a concept that multiple weak learners are trained to solve the problem. They are then combined to achieve better performance. The stack ensemble methods use various weak learners separately, and a meta-model is learned to predict outputs on top of all weak learners. In this paper, decision tree (DT) C4.5 was used as a weak learner, and selected features were used for training a meta DT classifier, which was created of stacked ensembled DT [74]. Each DT was tuned during training using a grid search. Tuned parameters contain the depth of the tree and feature importance metrics. Besides, for creating a stacked ensemble DT model, data are over-sampled using random sampling for overcoming an imbalance of the dataset issue [75]. The minority parts are trained with more weight on training each DT [76]. The performance of the system is evaluated using a three-fold cross-validation. The block diagram of the proposed method is depicted in Figure 1.

The demographic variables, and the questionnaires PCL-5, PTGI, MSPSS, PHQ-9, and PMH were used as the inputs of the prediction system, while the suicidal high and low-risk groups (calculated from SBQ-R questionnaire) were used as the binary output.

### 2.10. Validation

Three-fold cross-validation was used to guard against testing hypotheses suggested by the data (Type III errors) [77]. The following performance indices were calculated on each test fold as well as the cross-validated confusion matrix [78].

(3) $$\mathrm{Se}=\frac{\mathrm{TP}}{\mathrm{TP}+\mathrm{FN}}$$

(4) $$\mathrm{Sp}=\frac{\mathrm{TN}}{\mathrm{TN}+\mathrm{FP}}$$

(5) $$\mathrm{PPV}=\frac{\mathrm{TP}}{\mathrm{TP}+\mathrm{FP}}$$

(6) $$\mathrm{AUC}=\frac{\mathrm{Se}+\mathrm{Sp}}{2}$$

(7) $$\mathrm{MCC}=\frac{\left(\mathrm{TP}\times \mathrm{TN}-\mathrm{FP}\times \mathrm{FN}\right)}{\sqrt{\left(\left(\mathrm{TP}+\mathrm{FP}\right)\times \left(\mathrm{TP}+\mathrm{FN}\right)\times \left(\mathrm{TN}+\mathrm{FP}\right)\times \left(\mathrm{TN}+\mathrm{FN}\right)\right)}}$$

(8) $$K\left(C\right)=\frac{2\times \left(\mathrm{TP}\times \mathrm{TN}-\mathrm{FP}\times \mathrm{FN}\right)}{\left(\left(\mathrm{TP}+\mathrm{FP}\right)\times \left(\mathrm{FP}+\mathrm{TN}\right)+\left(\mathrm{TP}+\mathrm{FN}\right)\times \left(\mathrm{FN}+\mathrm{TN}\right)\right)}$$

(9) $$\mathrm{DOR}=\frac{\mathrm{TP}\times \mathrm{TN}}{\mathrm{FP}\times \mathrm{FN}}$$

(10) $$\mathrm{DP}=\left(\sqrt{\frac{3}{\pi }}\right)\times \log\left(\mathrm{DOR}\right)$$

where, TP, TN, FP, and FN were True Positives, True Negatives, False Positives, and False Negatives. Also, Se, Sp, and PPV are sensitivity, specificity, and positive predictive values (a.k.a., precision). The compositive indices AUC, MCC, K(C), DOR, and DP were the areas under the ROC Curve (a.k.a., the balanced diagnostic accuracy), the Matthews correlation coefficient [79], the Cohen’s Kappa coefficient [80], diagnostic odds ratio, and the discriminant power [81], respectively. Moreover, following the Standards for the Reporting of Diagnostic Accuracy Studies (STARD) guideline [82], the CI 95% of the performance indices of the cross-validated confusion matrix was presented. The selected features (i.e., demographic and questionnaire items) were also reported. Note that, since the accuracy is biased toward the majority class in an imbalanced dataset, it was not reported since it could overestimate the overall performance. Alternative objective indices such as AUC and MCC are preferred [79,81].

## 3. Results

The average age of all participants was *M* = 24.45 (*SD* = 6.651) years, and 72.1% were female. In addition, 63% were undergraduates, while 37% were graduate students. Furthermore, 60.7%, 5.8%, 23.6%, and 7.2% were liberal arts, basic sciences, engineering sciences, and medical sciences students. Among the 573 participants, 25% were at high-risk of suicide (SBQ-R ≥ 8). The characteristics of participants belonging to the low and high-risk groups are shown in Table 1 and further demographic information in Table A1. The relative frequency of the questionnaire items of the PCL, PTG, MSPSS, PHQ, PMH, and SBQ-R in low and high-risk groups are shown in Table A2, Table A3, Table A4, Table A5, Table A6 and Table A7. 

The selected features, along with their importance, are shown in Table 2. The weight parameter ranges from zero to one. The higher it is, the more important the feature is.

The performance of the prediction system is displayed in Table 3, where the performance indices are shown in each test fold, as well as the cross-validated confusion matrix. The CI 95% of the later indices are also provided.

The performance of the proposed diagnosis system could be provided based on the interpretations of the overall AUC, MCC, DP, and K(C) scores as the following. The proposed prediction system has excellent balanced diagnosis accuracy with a high correlation between predicted and observed class labels, fair discriminant power, and excellent class labeling agreement rate.

Moreover, a medical diagnosis system is clinically reliable if Se ≥ 80%, Sp ≥ 95% [83], PPV ≥ 95% [46], and DOR ≥ 100 [84]. The proposed diagnosis system fulfilled three conditions. However, the PVV is 94%. Thus, it is not entirely clinically reliable.

## 4. Discussion

To the best of our knowledge, this is the first study of its kind to make an accurate diagnosis of suicide ideation/behavior using robust ensemble learning in a university student sample in the MENA region. According to the results, we were able to identify 23 items out of 92 items that could be used to create a screening tool to distinguish young adults with high and low suicide ideation/behavior. The screening tool may have important implications for universities and policymakers that are responsible for university students’ suicide prevention and treatment. 

Based on our results, exposure to trauma, history of psychological illness, PTSD symptoms (9 items out of 20 items in PCL-5), positive mental health (6 items out of 9 in PMH), depression symptoms (3 items out of 9 in PHQ), post-traumatic growth (2 items out of 21 in PTGI), and social support (1 out of 12 items in the MSPSS) were recognized as the main variables for predicting suicide ideation and attempt.

The first important findings showed that exposure to trauma and PTSD symptoms could diagnose suicide ideation/behavior. The main items here were the type of exposure to trauma (first-hand or witnessing trauma) and PTSD symptoms. These findings complement previous findings showing that there is a strong link between trauma exposure and suicide risk [15,16,17,18,36,85,86]. PTSD symptoms such as cognitive distortion, irritability, and an intense feeling of distress when reminded of the traumatic event were the main variables that could predict suicide risk. Previous studies also found that PTSD has a strong and significant relationship with suicide ideation among young adults [87,88]. As Shafiei and colleagues [89] and Whiteman and colleagues [36] stated, people with traumatic experiences often face negative emotions such as anger, fear, horror, guilt, and shame. These negative emotions may decrease meaning in life, and, along with cognitive distortion, may decrease the ability to problem-solve and make decisions. 

Psychological illness is another input feature in three prediction systems that could predict suicide ideation/behavior. This is in line with previous studies that found a strong relationship between psychological distress and suicide ideation [85,90,91,92]. In our study, the main psychological problems that could predict suicide ideation/behavior were depression, anxiety, and obsessive-compulsive disorder. Previous studies also found that depression [13,41,86,93] and anxiety [13] predict suicide ideation. In line, feeling bad about oneself, lack of self-worth, and shame were selected by the proposed diagnostic system as another essential factor predicting suicide ideation/behavior. 

In our study, a lack of positive mental health, i.e., psychological and subjective well-being, was selected by the proposed diagnostic system, as another main predictor for suicide ideation/behavior. From the nine items of the PMH scale, six items were selected by the system as effective factors on suicide ideation/behavior. This is in agreement with some previous studies that have shown that positive mental health moderates the effects of stressful life events on suicidal thoughts [24]. In several recent studies, PMH is introduced as an essential target in suicide prevention and treatment programs because of its substantial impact on suicide ideation [26,27]. 

One of the notable factors in our research was post-traumatic growth (PTG) items. According to the literature, PTG moderates suicide risk among students who experienced traumatic events [32], and lower levels of PTG can increase suicidality [30]. In our study, two items from the PTG questionnaire were selected as predictors for suicidality, and both items are related to the appreciation of life. Accordingly, people who appreciate the value of their life and can appreciate each day are among the low-risk population for suicide ideation/behavior. This finding is consistent with previous studies that show having a reason to live [93] and gratitude [94] are protective factors for suicidality. 

Our system did not select social support as one of the well-studied buffering factors in the suicide literature [95,96] as a predictor of high vs. low suicide ideation/behavior. Although there was a significant correlation between social support’s items with some of the items of the PTGI and PMH, out of 12 items in the MSPSS, the only social support factor that was selected was the item: “I can talk about my problems with my family.” With regard to the idea that Iran is a collectivist society, and family and kinship have a strong influence on the individuals’ life, this finding is not surprising. For the majority of Iranians, the family is the first place to seek help and comfort. The role of parental connectedness and decreasing the risk of suicide is found in some other studies as well [7,97,98].

### 4.1. Limitations and Strength

The advantage of our study is the high accuracy of the proposed statistical model. It is the first study to analyze the comprehensive factors related to suicide ideation/behavior and to design a diagnostic system for university students at risk of suicide ideation/behavior in the MENA region. However, since a pure student sample was studied with the majority of the participants being female, one should refrain from generalizations to other samples. Furthermore, all variables were assessed using self-report measures. This is of great advantage with regard to the development of an easy-to-administer screening instrument. At the same time, bias effects cannot be ruled out. Therefore, it might be beneficial to cross-validate the described diagnostic model using interviewer-based assessments in future studies. Another limitation of the study is related to the distribution of gender and major among the sample. According to the Statistical Centre of Iran [99], the number of female students in all majors except the engineering field was higher than male students in 2016. Therefore, it was not surprising to have more female respondents than males in this study. The number of students in different majors, however, did not match with the country’s student population and we have more respondents from the fields of liberal arts and engineering than from basic sciences and medicine. We see this as the limitation of the study. However, the diagnostic system did not select these features and it is, thus, not problematic in the computer-aided-diagnostic systems. 

### 4.2. Implications

As mentioned before, the findings of this study have practical implications for policymakers and universities. Concerning the findings that trauma exposure and PTSD symptom were selected as the main variables in diagnosis of suicide ideation/behavior, routine assessment of cognitive distortion and PTSD symptoms among students at risk is important. The majority of the Iranian universities have free-of-charge counseling service. Having a trauma-informed counseling service to identify at-risk students is essential. As a lack of positive mental health and a lack of some of the post-traumatic growth’s items were selected as other predictors of suicide ideation/behavior, creating national programs to enhance PMH and PTG for the at-risk students is recommended. Based on the findings of this research, a screening tool will be designed, and the application can be used in universities to screen high-risk and low-risk students for suicide ideation/behavior for future prevention and intervention programs. Prospective studies should follow to further investigate the efficiency of the described screening tool.

## 5. Conclusions

To the best of our knowledge, this study is the first study to use robust ensemble learning to reliably and accurately diagnose suicide ideation/behavior among university students in the MENA region. Based on our results, we found that trauma exposure and PTSD symptoms, psychological problems (depression, anxiety, and obsessive-compulsive disorder), low mood, and low self-esteem can diagnose students that are at high risk for suicide ideation/behavior. We have also found that positive mental health can strongly affect suicide ideation/behavior, meaning that individuals that had high scores in PMH had less suicide ideation/behavior. People who appreciate life also showed less suicidality. Therefore, two items related to post-traumatic growth will be included in the final screening tool. Social support was only necessary for terms of family support in our prediction system. This study was performed to create a screening tool to identify university students at risk of suicide ideation/behavior and to help policymakers and universities to make appropriate on-time prevention and early intervention programs.

## Figures and Tables

**Table 1:** Demographic characteristics in MEAN ± SD [min-max] for interval, and frequency (percentage) for categorical variable.

Total (N = 573)		Low Risk (L.R) N = 430	High Risk (H.R) N = 143	*p*-Value ^1^
**Gender (Female)**		310 (72.1%)	109 (76.2%)	0.384
**Age (year)**		25.08 ± 6.8 [18–52]	22.4 ± 3.9 [19–43]	<0.001
**Grade**	Bachelor	253 (58.8%)	108 (75.5%)	0.008
	Masters	136 (31.6%)	22 (15.4%)	
	PhD	41 (9.5%)	13 (9.1%)	
**The field of study**	Liberal Arts	286 (62.3%)	80 (55.9%)	0.114
	Basic Sciences	22 (5.1%)	11 (7.7%)	
	Engineering Sciences	100 (23.3%)	35 (24.5%)	
	Medical Sciences	28 (6.5%)	13 (9.1%)	
	Foreign Languages	12 (2.8%)	4 (2.8%)	
**Educational level**	Weak	2 (0.5%)	7 (4.9%)	0.001
	Middle	82 (19.1%)	34 (23.8%)	
	Good	252 (58.6%)	75 (52.4%)	
	Excellent	93 (21.6%)	27 (189%)	
**Occupational status**	Unemployed	265 (61.6%)	105 (73.4%)	0.003
	Part time	99 (23%)	31 (21.7%)	
	Full time	66 (15.3%)	7 (4.9%)	
**Marital status**	Single	328 (76.3%)	126 (88.1%)	0.002
	Married	98 (22.8%)	16 (11.2%)	
	Divorced	3 (0.7%)	1 (0.7%)	
	Widow	1 (0.2%)	-	
**History of psychological illness**	None	342 (79.5%)	85 (59.4%)	0.001
	Bipolar	-	2 (1.4%)	
	Depression	25 (5.8%)	31 (21.7%)	
	Obsession	20 (4.7)	10 (7%)	
	Anxiety	43 (10%)	14 (9.8%)	
	Panic	-	1 (0.7%)	

^1^: The Mann-Whitney U Test was used for the age variable. For the categorical variables, the chi-square test was used. In the case of having less than five subjects in a cell, the Fisher’s exact test was used. The mode (median) was underlined in nominal (ordinal) measurement scales.

**Table 2:** The selected features and their importance in weight.

Index	Name	Weight	Index	Name	Weight
1	Exposure to Trauma	0.96	13	PMH 3	1
2	PCL 4	0.81	14	PMH6	0.91
3	PCL 5	0.72	15	PMH7	0.79
4	PCL 9	1	16	PMH8	0.97
5	PCL 10	0.86	17	PMH9	0/97
6	PCL 11	0.98	18	PHQ2	0.66
7	PCL 12	0.66	19	PHQ6	1
8	PCL 14	1	20	PHQ9	1
9	PCL 15	1	21	PTG2	1
10	PCL 16	0.61	22	PTG13	0.96
11	Psychological Illness	0.91	23	MSPSS8	0.72
12	PMH 2	1			

PCL: PTSD Symptomatology, PMH: Positive Mental Health Scale, PHQ: Patient health questionnaire, PTG: Post-traumatic Growth, MSPSS: Multidimensional Scale of Perceived Social Support.

**Table 3:** The performance indices of the proposed prediction system.

Indices Folds	TP	TN	FP	FN	Se (%)	Sp (%)	PPV (%)	AUC	MCC	DOR	DP	K(C)
1	39	143	1	9	81	99	98	0.90	0.86	620	2.73	0.85
2	40	139	4	8	83	97	91	0.90	0.83	174	2.19	0.83
3	37	140	3	10	79	98	93	0.88	0.81	173	2.19	0.81
Overall	116	422	8	27	81	98	94	0.90	0.83	227	2.30	0.83
CI 95%	-	-	-	-	[75–88]	[97–99]	[89–98]	[0.86–0.93]	[0.81–0.86]	[100–512]	[1.96–2.65]	[0.78–0.88]

TP: True Positives, TN: True Negatives, FP: False Positives, FN: False Negatives, Se: Sensitivity, Sp: Specificity, PPV: Positive Predictive value, AUC: Area under the ROC, MCC: Matthews Correlation Coefficient, DOR: Diagnostic Odds Ratio, DP: Discriminative Factor, K(C): Cohen’s Kappa.

## Appendix A

**Table A1:** Further demographic characteristics in frequency (percentage).

Total (N = 573)		(L.R) N = 430	(H.R) N = 143	*p*-Value ^1^
**Fathers’ job**	Unemployed	20 (4.7%)	3 (2.1%)	0.047
	Employed	87 (20.2%)	36 (25.2%)	
	Self-employed	174 (40.5%)	60 (42%)	
	Retired	149 (34.7%)	44 (30.8%)	
**Mothers’ job**	Housewife	334 (77.7%)	98 (68.5%)	0.023
	Employed	48 (11.2%)	26 (18.2%)	
	Self-employed	20 (4.7%)	4 (2.8%)	
	Retired	28 (6.5%)	15 (10.5%)	
**Economic status**	Weak	25 (5.8%)	3 (2.1%)	0.268
	Middle	227 (52.8%)	78 (54.5%)	
	Good	168 (39.1%)	61 (42.7%)	
	Excellent	10 (2.3%)	1 (0.7%)	
**Accommodation**	with family	341 (79.3%)	106 (74.1%)	0.195
	dormitory	89 (20.7%)	37 (25.9%)	
**History of physical illness**	None	364 (84.7%)	105 (73.4%)	0.003
	High blood pressure	2 (0.5%)	-	
	Cardiovascular	3 (0.7%)	7 (4.9%)	
	Musculoskeletal	9 (2.1%)	4 (2.8%)	
	Digestive	26 (6%)	7 (4.9%)	
	Diabetes	1 (0.2%)	3 (2.1%)	
	Hashimoto	2 (0.5%)	-	
	Hepatitis B	1 (0.2%)	-	
	Respiratory	5 (1.2%)	-	
	Kidney	1 (0.2%)	2 (1.4%)	
	Migraine	1 (0.2%)	3 (2.1%)	
	Obesity	1 (0.2%)	1 (0.7%)	
	Minor Thalassemia	2 (0.5%)	2 (1.4%)	
	Fauvism	-	1 (0.7%)	
	Visual impairment	1 (0.2%)	2 (1.4%)	
	Epilepsy	4 (0.9%)	-	
	Skin problem	1 (0.2%)	-	
	Cancer	3 (0.7%)	3 (2.1%)	
	Polycystic ovary syndrome	2 (0.5%)	3 (2.1%)	
	Genital issues	1 (0.2%)	-	
**History of using psychological medicine (Yes, %)**		31 (7.2%)	27 (18.9%)	<0.001
**History of using drugs, smoking, and alcohol (Yes, %)**		35 (8.1%)	26 (18.2%)	0.001
**Types of trauma (Yes, %)**	Sudden loss	154 (35.8%)	49 (34.3%)	0.7373
	Serious ill diagnosis	85 (19.8%)	29 (20.3%)	0.865
	Road accident	48 (11.2%)	10 (7%)	0.158
	Earthquake	8 (1.9%)	9 (6.3%)	0.007
	Loss of romantic relationship	105 (24.4%)	62 (43.4%)	<0.001
	Loss of important things	30 (7%)	9 (6.3%)	0.774
	Suicide	14 (3.3%)	5 (3.5%)	0.889
	Flood	2 (0.5%)	2 (1.4%)	0.557
	Sexual abuse	23 (5.3%)	20 (14%)	0.001
	Homicide	2 (0.5%)	1 (0.7%)	1.000
	Attack and violent	10 (2.3%)	11 (7.7%)	0.003
	Death treated	3 (0.7%)	2 (1.4%)	0.026
	Fire and explosion	4 (9%)	2 (1.4%)	1.000
	War and terror	6 (1.4%)	-	0.156
	Air accident	2 (0.5%)	1 (0.7%)	1.000
**How to experience trauma**	It happened to me directly.	177 (41.2%)	95 (66.4%)	<0.001
	I witnessed it.	105 (24.4%)	20 (14%)	
	It happened to a close person.	56 (13%)	8 (5.6%)	
	I was exposed to it as part of my job.	-	1 (0.7%)	

^1^ The chi-square test was used. In the case of having less than five subjects in a cell, the Fisher’s exact test was used. The mode (median) was underlined in nominal (ordinal) measurement scales.

**Table A2:** Frequency of the post-traumatic stress disorder (PTSD) Symptomatology (PCL-5) in low (L.R) and high-risk (H.R) groups (Cut-off = 31).

Q	Not at All		A Little Bit		Middle		Quite a Bit		Extremely		*p*-Value ^1^
	L.R	H.R	L.R	H.R	L.R	H.R	L.R	H.R	L.R	H.R	
1	52 (12.1%)	13 (9.1%)	129 (30%)	25 (17.5%)	107 (24.9%)	31 (21.7%)	97 (22.6%)	35 (24.5%)	44 (10.2%)	39 (27.3%)	<0.001
2	124 (28.8%)	29 (20.3%)	119 (27.7%)	30 (21%)	92 (21.4)	30 (21%)	66 (15.3%)	30 (21%)	29 (6.7%)	24 (16.8%)	<0.001
3	170 (39.5%)	29 (20.3%)	102 (23.7%)	28 (19.6%)	84 (19.5%)	26 (18.2%)	48 (11.2%)	35 (24.5%)	25 (5.8%)	25 (17.5%)	<0.001
4	97 (22.6%)	17 (11.9%)	115 (26.7%)	12 (8.4%)	106 (24.7%)	27 (18.9%)	78 (18.1)	50 (35%)	33 (7.7%)	37 (25.9%)	<0.001
5	198 (46%)	33 (23.1%)	110 (25.6%)	27 (18.9%)	66 (15.3%)	36 (25.2%)	39 (9.1%)	28 (19.6%)	16 (3.7%)	19 (13.3%)	<0.001
6	104 (24.2%)	19 (13.3%)	107 (24.9%)	32 (22.4%)	117 (27.2%)	35 (24.5%)	63 (14.7%)	34 (23.8%)	39 (9.1%)	23 (16.1%)	<0.001
7	127 (29.5%)	26 (18.2%)	99 (33%)	30 (21%)	108 (25.1%)	30 (21%)	63 (14.7%)	37 (25.9%)	33 (7.7%)	20 (14%)	<0.001
8	229 (53.3%)	64 (44.8%)	107 (24.9%)	38 (26.6%)	58 (13.5%)	25 (17.5%)	24 (5.6%)	12 (8.4%)	12 (2.8%)	4 (2.8%)	0.056
9	157 (36.5%)	18 (12.6)	94 (21.9%)	22 (15.4%)	86 (20%)	22 (15.4%)	58 (13.5%)	33 (23.1%)	34 (7.9%)	48 (38.6%)	<0.001
10	127 (29.5%)	19 (13.3%)	99 (23%)	19 (13.3%)	93 (21.6%)	22 (15.4%)	74 (17.2%)	37 (25.9%)	36 (8.4%)	46 (32.2%)	<0.001
11	126 (29.3%)	16 (11.2%)	119 (27.7%)	24 (16.8%)	82 (19.1%)	16 (11.2%)	69 (16%)	42 (29.4%)	33 (7.7%)	45 (315%)	<0.001
12	153 (35.6%)	24 (16.8%)	107 (24.9%)	18 (12.6%)	78 (18.1%)	37 (25.9%)	61 (14.2%)	33 (23.1%)	30 (7%)	31 (21.7%)	<0.001
13	200 (46.5%)	34 (23.8%)	106 (24.7%)	33 (23.1%)	69 (16%)	27 (18.9%)	45 (10.5%)	33 (23.1%)	10 (2.3%)	16 (11.2%)	<0.001
14	188 (43.7%)	29 (20.3%)	109 (25.3%)	27 (18.9%)	80 (18.6%)	23 (16.1%)	41 (9.5%)	32 (22.4%)	12 (2.8%)	32 (22.4%)	<0.001
15	185 (43%)	28 (19.6%)	122 (28.4%)	25 (17.5%)	69 (16%)	32 (22.4%)	39 (9.1%)	33 (23.1%)	15 (3.5%)	25 (17.5%)	<0.001
16	285 (66.3%)	61 (42.7%)	87 (20.2%)	31 (21.7%)	40 (9.3%)	24 (16.8%)	14 (3.3%)	18 (12.6%)	4 (0.9%)	9 (6.3%)	<0.001
17	145 (33.7%)	34 (23.8%)	133 (30.9%)	21 (14.7%)	83 (19.3%)	37 (25.9%)	44 (10.2%)	32 (22.4%)	25 (5.8%)	19 (13.3%)	<0.001
18	201 (46.7%)	44 (30.8)	123 (28.6%)	31 (21.7%)	55 (12.8%)	30 (21%)	37 (8.6%)	21 (14.7%)	14 (3.3%)	17 (11.9%)	<0.001
19	116 (27%)	18 (12.6%)	128 (29.8%)	22 (15.4%)	82 (19.1%)	35 (24.5%)	66 (15.3%)	41 (28.7%)	37 (8.6%)	27 (18.9%)	<0.001
20	165 (38.4%)	33 (22.4%)	113 (26.3%)	33 (22.4%)	69 (16%)	23 (16.1%)	48 (11.2%)	21 (14.7%)	35 (8.1%)	35 (24.5%)	<0.001

^1^: The Mann-Whitney U Test was used.

**Table A3:** Frequency of the Post-Traumatic Growth Inventory (PTGI) in low-risk (L.R) and high-risk (H.R) groups (Cut-off = 45).

Q	Not at All		A Little Bit		A Little		Middle		Quite a Bit		Extremely		*p*-Value ^1^
	L.R	H.R	L.R	H.R	L.R	H.R	L.R	H.R	L.R	H.R	L.R	H.R	
1	56 (13%)	23 (16.1%)	82 (19.1%)	14 (9.8%)	80 (18.6%)	22 (15.4%)	123 (28.6%)	41 (28.7%)	60 (14%)	25 (17.5%)	28 (6.5%)	18 (12.6%)	0.044
2	10 (2.3%)	25 (17.5%)	34 (7.9%)	22 (15.4%)	45 (10.5%)	29 (20.3%)	95 (22.1%)	28 (19.6%)	127 (29.5%)	27 (18.9%)	119 (27.7%)	12 (8.4%)	<0.001
3	34 (7.9%)	18 (12.6%)	42 (9.8%)	27 (18.9%)	62 (14.4%)	24 (16.8%)	128 (29.8%)	36 (25.2%)	92 (21.4%)	25 (17.5%)	72 (16.7%)	13 (9.1%)	<0.001
4	44 (10.2%)	36 (25.2%)	49 (11.4%)	31 (21.7%)	77 (17.9%)	30 (21%)	108 (25.1%)	24 (16.8%)	101 (23.5%)	11(.7%)	50 (11.6%)	11 (7.7%)	<0.001
5	47 (10.9%)	44 (30.8%)	49 (11.4%)	21 (14.7%)	76 (17.7%)	20 (14%)	94 (21.9%)	21 (14.7%)	69 (16%)	21 (14.7%)	95 (22.1%)	16 (11.2%)	<0.001
6	50 (11.6%)	41 (28.7%)	78 (18.1%)	39 (27.3%)	97 (22.6%)	21 (14.7%)	101 (23.5%)	22 (15.4%)	67 (15.6%)	16 (11.2%)	58 (13.5%)	14 (9.8%)	<0.001
7	28 (6.5%)	19 (3.3%)	38 (8.8%)	31 (21.7%)	81 (18.8%)	28 (19.6%)	132 (30.7%)	30 (21%)	93 (21.6%)	21 (14.7%)	37 (8.6%)	5 (3.5%)	<0.001
8	58 (13.5%)	45 (31.5%)	86 (20%)	29 (20.3%)	96 (22.3%)	39 (27.3%)	105 (24.4%)	17 (11.9%)	48 (11.2%)	8 (5.6%)	47 (10.9%)	8 (5.6%)	<0.001
9	51 (11.9%)	43 (30.1%)	88 (20.5%)	31 (21.7%)	84 (19.5%)	22 (15.4%)	87 (20.2%)	19 (13.3%)	72 (16.7%)	20 (14%)	47 (10.9%)	8 (5.6%)	<0.001
10	16 (3.7%)	10 (7%)	34 (7.9%)	21 (14.7%)	62 (14.4%)	32 (22.4%)	109 (25.3%)	42 (29.4%)	132 (30.7%)	23 (16.1%)	77 (17.9%)	15 (10.5%)	<0.001
11	19 (4.4%)	21 (14.7%)	46 (10.7%)	31 (21.7%)	71 (16.5%)	29 (20.3%)	118 (27.4%)	34 (23.8%)	100 (23.3%)	13 (9.1%)	76 (17.7%)	15 (10.5%)	<0.001
12	14 (3.3%)	10 (7%)	28 (6.5%)	16 (11.2%)	53 (12.3%)	27 (18.9%)	130 (30.2%)	41 (28.7%)	122 (28.4%)	29 (20.3%)	82 (19.1%)	20 (14%)	0.001
13	19 (4.4%)	26 (18.2%)	29 (6.7%)	25 (17.5%)	52 (12.1%)	34 (23.8%)	121 (28.1%)	24 (16.8%)	108 (25.1%)	17 (11.9%)	100 (23.3%)	17 (11.9%)	<0.001
14	33 (7.7%)	20 (14%)	39 (9.1%)	30 (21%)	66 (15.3%)	29 (20.3%)	113 (26.3%)	28 (19.6%)	101 (23.5%)	21 (14.7%)	78 (18.1%)	15 (10.5%)	<0.001
15	14 (3.3%)	12 (8.4%)	30 (7%)	19 (13.3%)	62 (14.4%)	22 (15.4%)	98 (22.8%)	27 (18.9%)	128 (29.8%)	36 (25.2%)	98 (22.8%)	27 (18.9%)	0.009
16	29 (6.7%)	25 (17.5%)	33 (7.7%)	27 (18.5%)	77 (17.9%)	24 (16.8%)	122 (28.4%)	28 (19.6%)	93 (21.6%)	24 (16.8%)	76 (17.7%)	15 (10.5%)	<0.001
17	19 (4.4%)	15 (10.5%)	39 (9.1%)	23 (16.1%)	57 (13.3%)	29 (20.3%)	119 (27.7%)	32 (22.4%)	126 (29.3%)	33 (23.1%)	69 (16%)	11 (7.7%)	<0.001
18	77 (17.9%)	58 (40.6%)	76 (17.7%)	24 (16.8%)	57 (13.3%)	21 (14.7%)	82 (19.1%)	15 (10.5%)	74 (17.2%)	13 (9.1%)	64 (14.9%)	12 (8.4%)	<0.001
19	22 (5.1%)	18 (12.6%)	28 (6.5%)	20 (14%)	52 (12.1%)	23 (16.1%)	100 (23.3%)	28 (19.6%)	133 (30.9%)	29 (20.3%)	95 (22.1%)	25 (17.5%)	<0.001
20	40 (9.3%)	28 (19.6%)	29 (6.7%)	14 (9.8%)	57 (13.3%)	18 (12.6%)	94 (21.9%)	28 (19.6%)	102 (23.7%)	330 (21%)	107 (24.9%)	25 (17.5%)	0.002
21	18 (4.2%)	14 (9.8%)	28 (6.5%)	17 (11.9%)	40 (9.3%)	19 (13.3%)	96 (22.3%)	28 (19.6%)	112 (26%)	30 (21%)	136 (31.6%)	35 (24.5%)	0.002

^1^: The Mann-Whitney U Test was used.

**Table A4:** Frequency of the Multidimensional Scale of Perceived Social Support (MSPSS) in low-risk (L.R.) and high-risk (H.R.) groups (Cut-off = 48).

Q	Very Strongly Disagree		Strongly Disagree		Mildly Disagree		Neutral		Mildly Agree		Strongly Agree		Very Strongly Agree		*p*-Value ^1^
	L.R	H.R	L.R	H.R	L.R	H.R	L.R	H.R	L.R	H.R	L.R	H.R	L.R	H.R	
1	26 (6%)	18 (12.6%)	34 (7.9%)	20 (14%)	40 (9.3%)	16 (11.2%)	66 (15.3%)	16 (11.2%)	85 (19.85%)	25 (17.5%)	76 (17.7%)	30 (21%)	103 (24%)	18 (12.6%)	0.001
2	30 (7%)	25 (17.5%)	22 (5.1%)	13 (9.1%)	29 (6.7%)	14 (9.8%)	50 (11.6%)	11 (7.7%)	81 (18.8%)	19 (13.3%)	89 (20.7%)	33 (23.1%)	129 (30%)	28 (19.6%)	0.001
3	12 (2.8%)	12 (8.4%)	12 (2.8%)	14 (9.8%)	25 (5.8%)	15 (10.5%)	53 (12.3%)	19 (13.3%)	79 (18.4%)	17 (18.9%)	80 (18.6%)	30 (21%)	168 (39.1%)	26 (18.2%)	<0.001
4	15 (3.5%)	17 (11.9%)	22 (5.1%)	15 (10.5%)	31 (7.2%)	13 (9.1%)	45 (10.5%)	20 (14%)	73 (17%)	28 (19.6%)	102 (23.7%)	30 (21%)	142 (33%)	20 (14%)	<0.001
5	35 (8.1%)	34 (23.8%)	24 (5.6%)	16 (11.2%)	31 (7.2%)	14 (9.8%)	51 (11.9%)	9 (6.3%)	63 (14.7%)	18 (12.6%)	64 (14.9%)	18 (12.6%)	162 (37.7%)	34 (23.8%)	<0.001
6	35 (8.1%)	15 (10.5%)	39 (9.1%)	20 (14%)	47 (10.9%)	32 (22.4%)	86 (20%)	15 (10.5%)	81 (18.8%)	27 (18.9%)	67 (15.6%)	14 (9.8%)	74 (17.2%)	20 (14%)	0.005
7	45 (10.5%)	28 (19.6%)	46 (10.7%)	27 (18.9%)	55 (12.8%)	20 (14%)	81 (18.8%)	14 (9.8%)	89 (20.7%)	26 (18.2%)	62 (14.4%)	13 (19.1%)	52 (12.1%)	15 (10.5%)	0.001
8	37 (8.6%)	30 (21%)	45 (10.5%)	30 (21%)	39 (9.1%)	20 (14%)	61 (14.2%)	17 (11.9%)	93 (21.6%)	26 (18.2%)	74 (17.2%)	13 (9.1%)	81 (18.8%)	7 (4.9%)	<0.001
9	35 (8.1%)	18 (12.6%)	34 (7.9%)	24 (16.8%)	36 (8.4%)	19 (13.3%)	67 (15.6%)	15 (10.5%)	80 (18.6%)	27 (18.9%)	85 (19.8%)	17 (11.9%)	93 (21.6%)	23 (16.1%)	<0.001
10	22 (5.1%)	17 (11.9%)	18 (4.2%)	18 (12.6%)	25 (5.8%)	11 (7.7%)	52 (12.1%)	16 (11.2%)	62 (14.4%)	25 (17.5%)	71 (16.5%)	19 (13.3%)	180 (41.9%)	37 (25.9%)	<0.001
11	17 (4%)	10 (7%)	15 (3.5%)	16 (11.2%)	22 (5.1%)	22 (15.4%)	60 (14%)	23 (16.1%)	92 (21.4%)	24 (16.8%)	101 (23.5%)	21 (14.7%)	123 (28.6%)	27 (18.9%)	<0.001
12	35 (8.1%)	25 (17.5%)	46 (10.7%)	22 (15.4%)	37 (8.6%)	20 (14%)	64 (14.9%)	14 (9.8%)	102 (23.7%)	23 (16.1%)	61 (14.2%)	21 (14.7%)	85 (19.8%)	18 (12.6%)	<0.001

^1^: The Mann-Whitney U Test was used.

**Table A5:** Frequency of the Patient health questionnaire (PHQ-9) in low-risk (L.R) and high-risk (H.R) groups (Cut-off = 10).

Q	Not at All		Several Days		More than Half the Days		Nearly Every Day		*p*-Value ^1^
	L.R	H.R	L.R	H.R	L.R	H.R	L.R	H.R	
1	96 (22.3%)	10 (7%)	171 (39.8%)	52 (36.4%)	121 (28.1%)	44 (30.8%)	42 (9.8%)	37 (25.9%)	<0.001
2	83 (19.3%)	7 (4.9%)	194 (45.1%)	44 (30.8%)	108 (25.1%)	52 (36.4%)	44 (10.2%)	40 (28%)	<0.001
3	170 (39.5%)	37 (25.9%)	120 (27.9%)	37 (25.9%)	78 (18.1%)	28 (19.6%)	62 (14.4%)	41 (28.7%)	<0.001
4	81 (18.8%)	10 (7%)	154 (35.8%)	33 (23.1%)	131 (30.5%)	43 (30.1%)	64 (14.9%)	57 (39.9%)	<0.001
5	187 (43.5%)	32 (22.4%)	122 (28.4%)	30 (21%)	85 (19.8%)	45 (31.5%)	35 (8.1%)	36 (25.2%)	<0.001
6	215 (50%)	27 (18.9%)	114 (26.5%)	33 (23.1%)	66 (15.3%)	41 (28.7%)	35 (8.1%)	42 (29.4%)	<0.001
7	175 (40.7%)	39 (27.3%)	135 (31.4%)	29 (20.3%)	84 (19.5%)	34 (23.8%)	36 (8.4%)	41 (28.7%)	<0.001
8	234 (54.4%)	52 (36.4%)	127 (29.5%)	33 (23.1%)	43 (10%)	29 (20.3%)	26 (6%)	29 (20.3%)	<0.001
9	347 (80.7%)	65 (45.5%)	55 (12.8%)	24 (16.8%)	21 (4.9%)	23 (16.1%)	6 (1.4%)	31 (21.7%)	<0.001

^1^: The Mann-Whitney U Test was used.

**Table A6:** Frequency of the Positive Mental Health Scale (PMH) in low-risk (L.R) and high-risk (H.R) groups (Cut-off = 15).

Q	Disagree		Mildly Disagree		Mildly Agree		Agree		*p*-Value ^1^
	L.R	H.R	L.R	H.R	L.R	H.R	L.R	H.R	
1	73 (17%)	45 (31.5%)	141 (32.8%)	52 (36.4%)	167 (38.8%)	36 (25.2%)	49 (11.4%)	10 (7%)	<0.001
2	19 (4.4%)	32 (22.4%)	91 (21.2%)	57 (39.9%)	200 (46.5%)	43 (30.1%)	120 (27.9%)	11 (7.7%)	<0.001
3	24 (5.6%)	35 (24.5%)	92 (21.4%)	50 (35%)	183 (42.6%)	49 (34.3%)	131 (30.5%)	9 (6.3%)	<0.001
4	41 (9.5%)	39 (27.3%)	100 (23.3%)	40 (28%)	182 (42.3%)	49 (34.3%)	107 (24.9%)	15 (10.5%)	<0.001
5	27 (6.3%)	29 (20.3%)	94 (21.9%)	45 (31.5%)	209 (48.6%)	58 (40.6%)	100 (23.3%)	11 (7.7%)	<0.001
6	28 (6.5%)	38 (26.6%)	107 (24.9%)	48 (33.6%)	179 (41.6%)	45 (31.5%)	116 (27%)	12 (8.4%)	<0.001
7	32 (7.4%)	41 (28.7%)	96 (22.3%)	42 (29.4%)	214 (49.8%)	48 (33.6%)	88 (20.5%)	12 (8.4%)	<0.001
8	20 (4.7%)	35 (24.5%)	113 (26.3%)	51 (35.7%)	196 (45.6%)	47 (32.9%)	101 (23.5%)	10 (7%)	<0.001
9	18 (4.2%)	32 (22.4%)	63 (14.7%)	41 (28.7%)	221 (51.4%)	51 (35.7%)	128 (29.8%)	19 (13.3%)	<0.001

^1^: The Mann-Whitney U Test was used.

**Table A7:** Frequency of the Suicide Behaviors Questionnaire-Revised (SBQ-R) in low-risk (L.R) and high-risk (H.R) groups (Cut-off = 8, for the first question is 2).

Q		L.R	H.R
1	Never	322 (74.9%)	2 (1.4%)
	It was just a brief passing thought	102 (23.7%)	60 (42%)
	I have had a plan at least once to kill myself but did not try to do it.	6 (1.4%)	37 (25.9%)
	I have had a plan at least once to kill myself and really wanted to die.	-	23 (16.1%)
	I have attempted to kill myself but did not want to die.	-	11 (7.7%)
	I have attempted to kill myself and really hoped to die.	-	10 (7%)
2	Never	385 (89.5%)	22 (15.4%)
	Rarely (1 time)	41 (9.5%)	47 (32.5%)
	Sometimes (2 times)	4 (0.9%)	36 (25.2%)
	Often (3–4 times)	-	22 (15.4%)
	Very often (5 or more times)	-	16 (11.2%)
3	No	385 (89.5%)	53 (37.1%)
	Yes, at one time, but did not really want to die	40 (9.3%)	36 (25.2%)
	Yes, at one time, and really wanted to die	3 (0.7%)	21 (14.7%)
	Yes, more than once, but did not want to do it	2 (0.5%)	17 (11.9%)
	Yes, more than once, and really wanted to do it	-	16 (11.2%)
4	Never	346 (80.5%)	12 (8.4%)
	No chance at all	44 (10.2%)	22 (15.4%)
	Rather unlikely	32 (7.4%)	45 (31.5%)
	Unlikely	5 (1.2%)	23 (16.1%)
	Likely	3 (0.7%)	32 (22.4%)
	Rather likely	-	7 (4.9%)
	Very likely	-	2 (1.4%)
